# Clusters of amniotic fluid cells and their associated early neuroepithelial markers in experimental myelomeningocele: Correlation with astrogliosis

**DOI:** 10.1371/journal.pone.0174625

**Published:** 2017-03-30

**Authors:** Jolanta Zieba, Amanda Miller, Oleg Gordiienko, George M. Smith, Barbara Krynska

**Affiliations:** Shriners Hospitals Pediatric Research Center, Center for Neural Repair and Rehabilitation, Lewis Katz School of Medicine at Temple University, Philadelphia, Pennsylvania, United States of America; Instituto Butantan, BRAZIL

## Abstract

Myelomeningocele (MMC) is the most common and severe disabling type of spina bifida resulting in the exposure of vulnerable spinal cord to the hostile intrauterine environment. In this study, we sought to examine the cellular content of fetal amniotic fluid (AF) in MMC and explore a correlation between these cells and pathological development of MMC. MMC was induced in fetal rats by exposing pregnant mothers to all-trans retinoic acid and AF samples were collected before term. Cells were isolated from AF samples and morphologically and phenotypically characterized in short-term cultures. In addition, the spinal cord injury in MMC fetuses was assessed by immunohistochemical examination of astrogliosis. We identified a population of cells from the AF of MMC fetuses (MMC-AF) that formed adherent clusters of tightly packed cells, which were absent from the AF of normal control fetuses (norm-AF). MMC-AF clusters contained cells co-expressing adherens junction associated proteins (ZO-1), N-cadherin and F-actin at sites of cell-cell contacts. In addition, they expressed markers of early neuroepithelial cells such as SOX-1 and Pax-6 along with other stem/progenitor cell markers such as SOX-2 and nestin. Subpopulations of cells in MMC-AF clusters also expressed more advanced differentiation markers such as doublecortin and GFAP. We found that the appearance of cluster forming cells in cultures from MMC-AF correlated with activation of astrogliosis associated with the spinal cord injury in MMC fetuses. In summary, we identified a neuroepithelial cell population in the AF of MMC fetuses that formed adherent clusters in culture and we characterized cellular markers of these cells. Our data suggests that the phase of the disease is a crucial factor in the emergence of these cells into the AF and that these cells may provide a new and important platform for studying the progression of MMC and development of improved strategies for the repair and diagnosis of MMC prenatally.

## Introduction

Myelomeningocele (MMC), the most common and severe form of spina bifida, is a devastating congenital defect. [1,2]. It is characterized by protrusion of the meninges and spinal cord through the overlying vertebral defect and wound opening in the skin [3]. Children affected by MMC face significant and life-long physical disabilities including leg paralysis, sensory loss, bowel and bladder dysfunctions, skeletal deformations, and Arnold-Chiari II malformation with secondary hydrocephalus often requiring lifelong support and institutional care [4–6]. The etiology in most cases of MMC is multifactorial involving teratogenic, genetic and nutritional factors [7–9]. In particular, folic acid deficiency has been implicated in increased risk of neural tube defects, including MMC [10,11]. However, despite mandatory folate supplementation and routine treatment of women with folic acid before or during early pregnancy, neural tube defects remain among the most common congenital abnormalities in humans. Treatment and management of patients with these defects continues to have a huge economic burden on the health care system [12,13].

The pathogenesis of MMC is not well understood, but growing evidence indicates that secondary *in utero* damage to the exposed spinal cord during the later phase of gestation is associated with loss of neurological function in fetuses with MMC [14–17]. The classical treatment for MMC consists of surgical closure of the MMC defect soon after birth, but these children usually require lifelong support, rehabilitation, and institutional care [18,19]. In recent years, intrauterine surgical closure of the MMC defect has developed as a strategy to minimize spinal cord damage before birth. A multicenter randomized trial showed that prenatal surgical closure was more successful in restoration of neurological function than postnatal, however, the surgical procedure can only be performed in a fraction of patients and restoration of neurological function is limited in many children [20,21]. As an alternative to surgical intervention, tissue engineering has emerged as a regenerative strategy for the prenatal treatment of MMC defects [22,23]. Thus, a definitive and early diagnosis of MMC is important for any prenatal treatment of patients with MMC. However, diagnosis of an open neural tube defect e.g., MMC during early gestation, can be difficult and patient selection for an appropriate intervention remains challenging [20].

During gestation, amniotic fluid constitutes an important part of fetal environment and provides a source of cells for the prenatal diagnosis or therapy of developmental defects [24]. Although neural cells have been identified in the AF of fetuses with neural tube defects [25–28], a more comprehensive analysis of AF cell phenotypic profiling during the development of these defects has only recently started to be performed [29–31].

In this study, we sought to explore changes in the cellular content of MMC-AF in relationship to the pathological development of MMC. A better understanding of this relationship would be helpful in developing novel strategies for prenatal treatment and/or diagnosis of MMC. For this study, MMC was established using the retinoic acid-induced rat model, which is developmentally and anatomically analogous to human MMC and provides an excellent translational model for studying MMC in rats [32,33].

## Materials and methods

All animal studies in this project were performed under protocols approved by Temple University's Institutional Animal Care and Use Committee (IACUC). The Temple University IACUC functions to ensure compliance with all federal and state regulations on the humane care and use of animals in research, including the provisions of the Animal Welfare Act and the PHS Policy on Humane Care and Use of Laboratory Animals. All methods of euthanasia were consistent with the recommendations of the Panel on Euthanasia of the American Veterinary Medical Association (AVMA).

### Retinoic acid-induced animal model of MMC

All experiments were conducted under the guidelines of Temple University’s IACUC and the National Institutes of Health Guide for Care and Use of Laboratory Animals. Time-dated Sprague-Dawley pregnant rats obtained from Charles River Laboratories (Wilmington, MA) were placed on a standard dark: light schedule. An MMC defect was induced in the fetuses of time-dated pregnant rats by gavage of a single dose of 50 mg/kg of all trans-retinoic acid (Sigma-Aldrich Chemical, Saint Louis, MO) dissolved in olive oil on embryonic day 10 (E10), as described previously [32,34]. Normal control mothers were gavage fed with olive oil. A total of 38 dams were used in these studies: 30 time-dated pregnant dams were gavage fed with all trans-retinoic acid and 8 were exposed to olive oil alone. AF samples from normal fetuses (n = 97) and MMC fetuses (n = 201) for isolation of AF cells were collected between E18 and E21 (term = E22). Dams were euthanized using chamber inhaled CO_2_, and the total number of fetuses were determined following the midline laparotomy and exposure of the uterus. AF samples were collected using an 18-guage regular bevel needle (Becton, Dickinson and Company, USA) on 1 ml syringe (Becton, Dickinson and Company, USA) under aseptic conditions into 1.5 ml Eppendorf tubes (Eppendorf, USA). After collection of AF, the uterus and gestational membranes were removed and each fetus was examined for the presence of lumbosacral MMC defect. The incidence of isolated MMC defects was observed in 62.8% (201/320) of fetuses. After harvesting, fetuses were collected and euthanized by decapitation according to standard procedures.

### Isolation of cluster-forming cells

AF samples from MMC fetuses and normal controls were centrifuged at 3,000 rpm for 10 min. Supernatants of AF were removed. The cell pellets from each AF sample were then gently re-suspended in 0.5–1 ml of standard growth media containing DMEM (Life Technologies, USA) supplemented with 10% fetal bovine serum (FBS) (Atlanta Biologicals, USA), penicillin 100 U/mL /streptomycin 100 μg/mL (Mediatech Inc.), which supports the growth of cells in adherent cultures without any feeder layer and after gentle mixing 0.25 ml of cell suspension was plated per 1 well of 4-well plastic dish (Thermo Fisher Scientific, USA) with glass cover slips (Carolina Biological Supply Company, USA) and placed in a 95% humidified, 5% CO_2_ incubator at 37°C. Cells remained in culture for 24–72 hours. At that time, the cells derived from all AF samples underwent morphological and phenotypical analyses. To that end, cells were first inspected for morphological appearance with an inverted microscope and photographs were taken using a Nikon Eclipse E2000 microscope (Nikon USA, Melville, NY) and ACT-1 software (Nikon USA). Cultures were then fixed and stained with 1% methylene blue or fixed with 4% paraformaldehyde and examined by immunocytochemistry.

### Isolation of mesenchymal cells

Primary cultures of mesenchymal cells were obtained from AF as previously described [35]. In brief, for the isolation of AF-derived population of mesenchymal cells, AF samples obtained from normal rat dams on E21 were centrifuged at 3,000 rpm for 10 min. After centrifugation, cells were resuspended in culture media containing DMEM (Life Technologies, USA) supplemented with 20% fetal bovine serum (FBS) (Atlanta Biologicals), penicillin 100 U/mL /streptomycin 100 μg/mL (Mediatech Inc.) and the cells were plated at the density of 150 K cells per one well of 6 well dish. Cultures were inspected for cell attachment after 48 hours, then the non-adherent cells and debris were removed and the media replaced. After the cells grew to near confluency, they were harvested using 0.25% trypsin (Gibco by life Technologies, USA) and plated at the density of 10,000 cells per cm^2^ in culture media for expansion. To confirm the mesenchymal phenotype, cells were examined by immunocytochemistry for expression of mesenchymal markers including vimentin (mouse monoclonal, 1:200 dilution, Dako), fibronectin (rabbit polyclonal, 1:500 dilution, Dako) and CD44 (mouse monoclonal, 1:200 dilution, BD Pharmingen). A mouse monoclonal antibody directed against pan-Cytokeratin (mouse monoclonal, 1:200 dilution, Santa Cruz Biotechnology) was used to exclude the presence of epithelial cells. Passage two cells were frozen for use in future experiments as negative controls for neural cell markers.

### Tissue processing and immunofluorescence

For histological analyses, fetal rats were collected as described above and then fixed in 10% neutral buffer formalin at 4°C for 3 days. After fixation, samples were equilibrated in 30% sucrose in phosphate buffer at 4°C before embedding in OCT (Sakura Finetek USA, USA), and frozen. Serial 15 μm coronal sections were obtained through the center of the MMC defect and mounted on charged glass slides (Superfrost Plus, Fisher Scientific, USA). Sections assigned for immunofluorescence were immersed in 0.01 mol/L sodium citrate buffer, 0.05% Tween 20, pH 6.0, heated in a microwave, and rinsed in 1X phosphate buffered saline (PBS) solution before blocking nonspecific antibody reactions with 5% normal goat serum in 1% bovine serum albumin/PBS solution for 45 minutes at room temperature. Sections were then incubated with a rabbit polyclonal antibody directed against GFAP (1:500 dilution, DAKO) and a mouse monoclonal antibody directed against nestin (1:200 dilution, Chemicon International) in 1% bovine serum albumin/PBS solution overnight at 4°C. On the next day, sections were washed with 1X PBS solution, followed by incubation with anti-rabbit AlexaFluor 555 and anti-mouse AlexaFluor 488 secondary antibody (1:1000 dilution, Life Technologies, USA) in 1% bovine serum albumin/PBS solution for 1 hour at room temperature in the dark. Slides were then mounted with a ProLong Gold anti-fade reagent (ProLong Gold, Life Technologies, USA), coverslipped, and examined using a Nikon Eclipse 80i fluorescence microscope (Nikon USA, Melville, NY). Imaging was performed using a CoolSNAP EZ CCD camera and NIS-elements software (Nikon USA). The exposure time and image acquisition settings in each channel were kept constant for all analyzed sections. The images were merged using NIS-elements software (Nikon USA) and visually estimated for the presence of GFAP and/or nestin positive cells. All analyses were made using cross sections obtained from the lumbar spinal cord region of three fetuses per age group by examining at least three sections from each fetus.

### Immunocytochemistry

Immunofluorescence was carried out according to standard techniques. Cells were fixed in 4% paraformaldehyde in PBS for 10 minutes and then washed in 1X PBS. For immunostaining with primary antibodies against SOX-1, SOX-2, Pax-6, nestin, GFAP, and doublecortin, cells were permeabilized with 0.3% Triton X-100 (Sigma) in 1X PBS for 12 min at room temperature while shaking and subsequently blocked with 10% goat or donkey serum in 1X PBS for 1 hour at room temperature. For immunostaining with primary antibodies against ZO-1 and N-cadherin, cells were exposed to 0.5% Triton X-100 with 10% goat serum in 1X PBS for 2 hours at room temperature while shaking. Cells were then incubated with primary antibodies overnight at 4°C. Cellular markers were studied with mouse monoclonal antibodies directed against nestin (1:200 dilution, Chemicon International), GFAP (1:200 dilution, Sigma-Aldrich), N-cadherin (1:200 dilution, BD Transduction Laboratories), rabbit polyclonal antibodies directed against SOX-1 (1:500 dilution, Cell Signaling Technology), SOX-2 (1:500 dilution, Santa Cruz Biotechnology), Pax-6 (1:500 dilution, Biolegend), GFAP (1:500 dilution, DAKO), ZO-1 (1:500 dilution, Thermo Fisher Scientific) and goat polyclonal antibody directed against doublecortin (1:500 dilution, Santa Cruz Biotechnology). All primary antibodies were previously validated for use in rats. For negative controls, immunostaining was performed without primary antibodies. Species-specific Alexa Fluor 555 or 488 secondary antibodies (1:1000 dilution, Life Technologies, USA) were added and incubated for 1 hour at room temperature in the dark. Slides were then counterstained with DAPI, washed with 1X PBS, mounted with a ProLong Gold anti-fade reagent (ProLong Gold, Life Technologies, USA), coverslipped, and examined using a Nikon Eclipse 80i fluorescence microscope (Nikon USA, Melville, NY). Imaging was performed using a CoolSNAP EZ CCD camera and NIS-elements software (Nikon USA). Mesenchymal cells obtained from the AF of pregnant dams that were not exposed to retinoic acid treatment were used as negative controls for neural cell markers.

### Cell counting

Cluster-forming cells were isolated as described above. For quantification of GFAP positive cells, at 48 hours in culture cell clusters were manually collected using an inverted bright-field microscope. Individual clusters were dissociated into single cells and plated in culture media onto glass coverslips. After 24 hours cells were fixed in 4% paraformaldehyde and examined by immunocytochemistry for GFAP expression and counterstained for DAPI as described above. Immunoassayed cells were analyzed using ImageJ software (NIH Image, NIH Bethesda, USA). Quantitative analyses were performed by counting at least 300 cells per cluster/coverslip. Cells were counted from 15 clusters/coverslips from three independent cultures. The proportion of GFAP positive cells within each cluster was expressed as a ratio of GFAP positive cells to the total number of cells visualized by DAPI and data are presented as a range of values.

### Cluster counting

For counting of cell clusters expressing ZO-1 and N-cadherin, randomly selected cultures were assessed for ZO-1 and N-cadherin expression as described above. Immunoassayed cells were examined using a Nikon Eclipse 80i fluorescence microscope and NIS elements software (Nikon USA). Quantitative analyses were performed by counting all clusters of cells visualized by DAPI and clusters with junctional double staining for ZO-1 and N-cadherin on each coverslip. The number of clusters with strong ZO-1 and N-cadherin junctional signal was averaged from 6 coverslips from 3 independent cultures and is presented as the average percent of cell clusters.

### Fluorescence staining for ZO-1 and F-actin

Cells were fixed in 4% paraformaldehyde in PBS for 15 min, exposed to 0.5% Triton X-100 with 10% goat serum in 1X PBS for 2 hours at room temperature while shaking. The fixed cells were then incubated overnight with a rabbit polyclonal antibody directed against ZO-1 (1:500 dilution, Thermo Fisher Scientific) at 4°C while shaking. Cells were then washed and a secondary antibody conjugated to AlexaFluor 555 (1:1000, dilution, Life Technologies, USA) was added for 1 hour at room temperature in the dark. After that cells were washed with 1X PBS and Alexa Fluor 488 Phalloidin (A12379, 1:40 dilution, Life Technologies, USA) was used for staining F-actin. To that end, cells were incubated for 20 minutes at room temperature with Alexa Fluor 488 Phalloidin at 1:40 dilution and then washed with 1X PBS. Slides were then counterstained with DAPI, washed with 1X PBS, mounted with a ProLong Gold anti-fade reagent (ProLong Gold, Life Technologies, USA), coverslipped, and examined using a Nikon Eclipse 80i fluorescence microscope (Nikon USA, Melville, NY). Imaging was performed using a CoolSNAP EZ CCD camera and NIS-elements software (Nikon USA).

## Results

### Morphological characterization of MMC-AF cells

**Fig 1:**
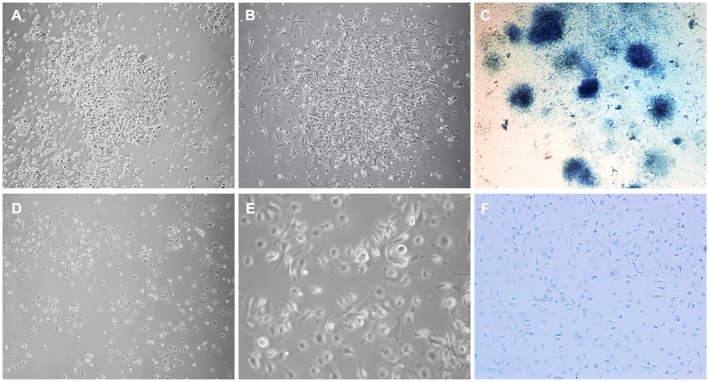
Comparison of morphological appearance of amniotic fluid cells from MMC and normal fetuses. Representative phase-contrast micrographs of cells isolated from MMC-AF at E21 illustrate clusters of densely packed amniotic fluid cells (A) at 24 hours after plating, (B) at 72 hours after plating, and (C) image of distinct clusters of cells stained with methylene blue at 72 h after plating. In contrast, representative micrographs illustrate a loose monolayer growth pattern of amniotic fluid cells isolated from age-matched normal controls shown at (D) lower and (E) higher magnification, and (F) image of these cells stained with methylene blue at 72 h after plating showing the absence of cell clusters (compare F and C). (Magnification A, B, D, F10X; E20 X; C4X).

We evaluated the behavior and morphology of cells isolated from MMC-AF between E20 and E21 (Fig 1A–1C) and those of age-matched controls isolated from norm-AF in short–term cultures (Fig 1D–1F). Previous studies show cells cultured from AF in cases of neural tube defects adhere rapidly to plastic or glass surfaces [25,26]. Therefore, we monitored the attachment and growth of MMC-AF cells and norm-AF controls after plating in culture. We observed that MMC-AF cells adhered in less than 24 hours after plating (Fig 1A) while cells isolated from norm-AF adhered to the tissue culture surface relatively slowly, taking about 48–72 hours (Fig 1D and 1E). 72 hours after plating, microscopic analysis of MMC-AF cells revealed that these cells grew primarily in distinct clusters of densely packed cells with compact morphology and morphologically heterogeneous cells typically at the cluster periphery (Fig 1B). Methylene blue staining demonstrated attached cell clusters of varying sizes in MMC-AF cultures at 72 h after plating (Fig 1C). In contrast, cells derived from norm-AF did not grow in cell clusters as observed in the MMC-AF group (Fig 1F). Norm-AF cells formed a loose monolayer of morphologically heterogeneous cells differing in shape and size composed of a majority of elongated fibroblast-like cells and a smaller fraction of spindle-shape cells typical for mesenchymal cells (Fig 1D and 1E). The distinct difference in the initial cell behavior indicated differences in the underlying cell populations. We then characterized the MMC-AF cell clusters to better understand MMC associated changes in the cellular content of AF.

### Adherens junctions in clusters of MMC-AF cells

**Fig 2:**
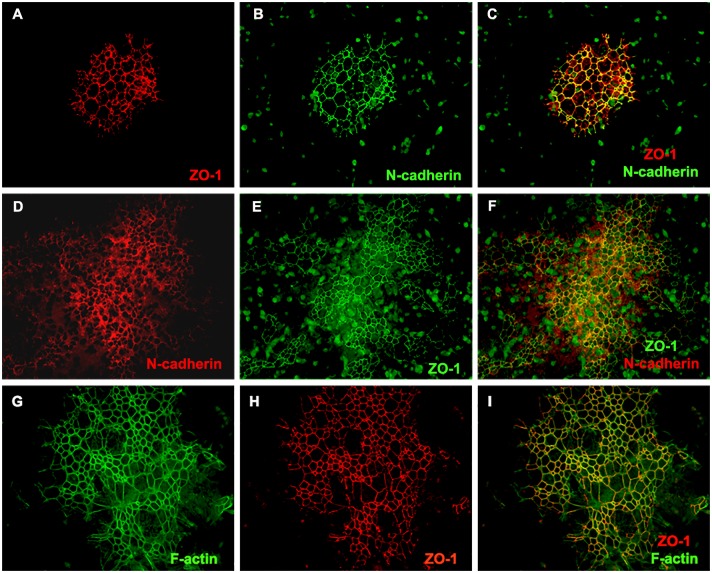
Expression of N-cadherin, ZO-1, and the distribution of F- actin in amniotic fluid cells of MMC fetuses. (A and B) Amniotic fluid cells were stained 72 hours after isolation with antibodies directed against ZO-1, N-cadherin or with Alexa Fluor 488 Phalloidin for staining F-actin. Double immunofluorescence staining with antibodies to ZO-1 and N-cadherin in small MMC-AF clusters. (C) Photomicrograph of a merged image of (A) and (B) demonstrates co-localization and uniform distribution of ZO-1 and N-cadherin. (D and E) Double immunofluorescence staining with antibodies to N-cadherin and ZO-1 in large MMC-AF clusters. (F) Photomicrograph of a merged image of (D) and (E) demonstrates co-localization of ZO-1 and N-cadherin with areas of stronger and weaker expression. Note that, high ZO-1 expression was generally correlated to high N-cadherin expression. (G and H) Representative photomicrographs demonstrate distribution of F-actin and ZO-1. (I) Photomicrograph of a merged image of (G) and (H) showing co-localization of ZO-1 and F- actin in amniotic fluid cells (Magnification 20 X).

In order to characterize MMC-AF cells, which grew in densely packed clusters, we examined the expression of adherens junction proteins that could play a role in clustering of these cells. The adherens junction proteins ZO-1 and N-cadherin that contribute to cell-cell adhesion in neuroepithelium [36], were detected at the cell-cell contacts of MMC-AF cells (Fig 2A–2F). Analysis of ZO-1 and N-cadherin distribution revealed that in 18.8 [± 9.8%] of all clusters, ZO-1 and N-cadherin exhibited a uniform and overlapping distribution and showed strong staining at sites of cell-cell contact [Fig 2A–2C]. These clusters were usually smaller in size. The vast majority of MMC-AF clusters contained areas of strong junctional staining in areas of densely clustered cells. In less dense areas, ZO-1 and N-cadherin expression levels were lower or virtually lost indicating a downregulation of these proteins most likely related to differentiation of these cells [Fig 2D–2F]. Adherens junctions are protein complexes linked to a ring-like cytoskeleton of actin microfilaments [F-actin] [37]. In order to characterize the distribution of F-actin, MMC-AF cells were stained with fluorescein-conjugated Phalloidin that binds specifically to F-actin [38]. F-actin was observed along the boundaries between adjacent cells and co-localized with ZO-1 (Fig 2G–2I). In cells lacking ZO-1 immunoreactivity, the linear distribution of F-actin along cell-cell borders was absent (Fig 2G–2I), suggesting reorganization of the actin cytoskeleton in cells undergoing differentiation. Tight junction proteins such as occludin and E-cadherin were not detected in these cells (data not shown). These findings indicate that MMC-AF cells grow in dense clusters and form ZO-1 and N-cadherin-based adherens junctions, which is the feature of neuroepithelium.

### Phenotypic characterization of cells growing in MMC-AF clusters

**Fig 3:**
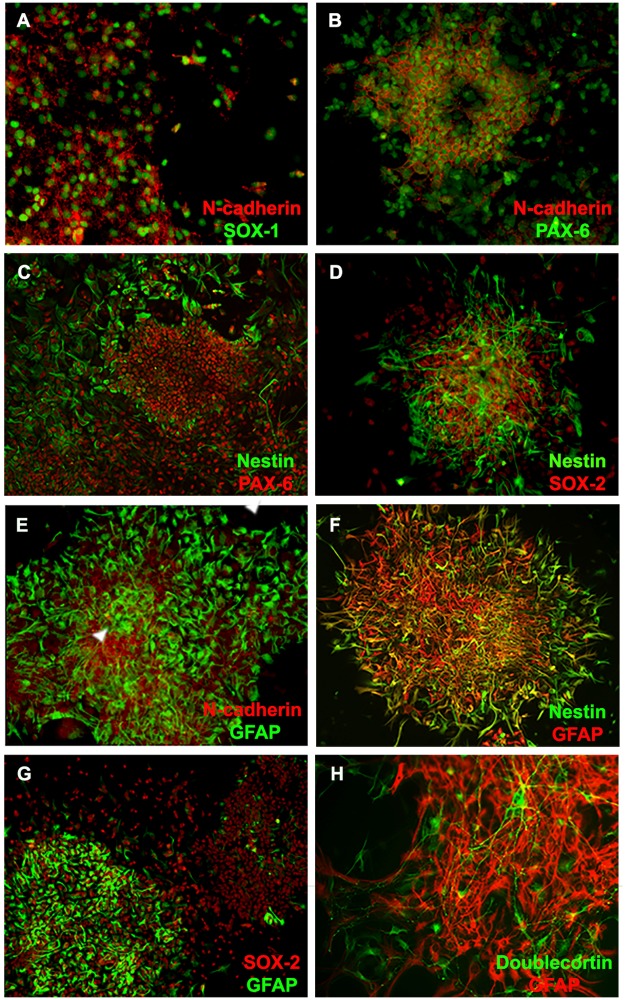
Expression of neural markers by amniotic fluid cells isolated from MMC. (A-D) Double immunofluorescence staining demonstrates that the majority of cells within N-cadherin positive clusters stained positive for markers of early neuroepithelial cells such as SOX-1 and Pax-6 along with other progenitor markers such as nestin and SOX-2. (E and F) Double immunofluorescence staining for the expression of N-cadherin and GFAP show N-cadherin-positive clusters with areas of strong junctional expression of this protein (arrowhead) contained GFAP positive cells. (G) Double immunofluorescence staining for the expression of GFAP and Nestin shows GFAP positive cells, which often co-expressed nestin as wells as GFAP positive cells that were negative for nestin. (H) Double immunofluorescence staining for the expression of GFAP and SOX-2 demonstrates clusters with high and low fraction of GFAP positive cells. (I) GFAP positive cells within clusters were also accompanied by doublecortin positive neurons. (Magnification 20 X).

Since cells in these clusters showed features of neuroepithelium, we then phenotypically characterized them and found that the majority of cells within MMC-AF clusters stained positive for SOX-1 and Pax-6, markers of early neuroepithelial cells, along with the neural progenitor cell markers, nestin and SOX-2 (Fig 3A–3D). Although, we did not quantify the exact proportion of cells expressing these markers due to dense clusters of cells often forming double or triple layers, they were present in all clusters seen in MMC-AF cultures. SOX-1 and Pax-6 positive cells were associated with N-cadherin-rich areas (Fig 3A and 3B). SOX-1 and N-cadherin positive cells were found mainly within clusters, while Pax-6 was found both within clusters and at their periphery (Fig 3B and 3C). This may suggest the transition of early neuroepithelial cells into progenitors that migrate away from dense clusters of undifferentiated cells similar to the migration of differentiating cells from the germinal zone [39]. In order to determine the presence of immature neurons and glia in MMC-AF clusters, we examined the expression of more advance differentiation markers. We found that MMC-AF clusters contained a population of GFAP expressing cells (Fig 3E). GFAP expressing cells were distributed throughout these clusters and often co-expressed nestin, which is transiently expressed during early astrocyte development, indicating their immature phenotype (Fig 3F). We also found a number of GFAP expressing cells that were negative for nestin and nestin expressing cells that did not show GFAP immunoreactivity (Fig 3F). Although the percentage of GFAP positive cells found within clusters ranged from 10.1% to 80.2% (Fig 3G), all clusters contained GFAP expressing cells and the vast majority of clusters had large sub-population of GFAP positive cells (Fig 3E, 3F and 3G). This observation was also accompanied by the presence of doublecortin positive cells, a marker of immature neurons (Fig 3I). Doublecortin positive cells were generally found at the periphery of MMC-AF clusters and were negative for GFAP (Fig 3I). Taken together, these findings suggest that MMC-AF contains a population of early neuroepithelial cells at various maturation stages that exist in clusters -like formation.

### MMC-AF clusters and astrogliosis in fetal rats with MMC

**Fig 4:**
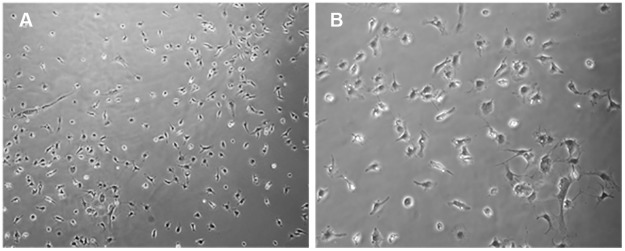
A monolayer of amniotic fluid cells from MMC fetuses at E19. (A and B) At 72 h after plating, representative phase-contrast micrographs of amniotic fluid cells illustrate a monolayer growth pattern of amniotic fluid cells in the absence of cell clusters in cultures of amniotic fluid cells isolated from MMC-AF obtained at E19. (Magnification (A 10 X; B20 X).

**Fig 5:**
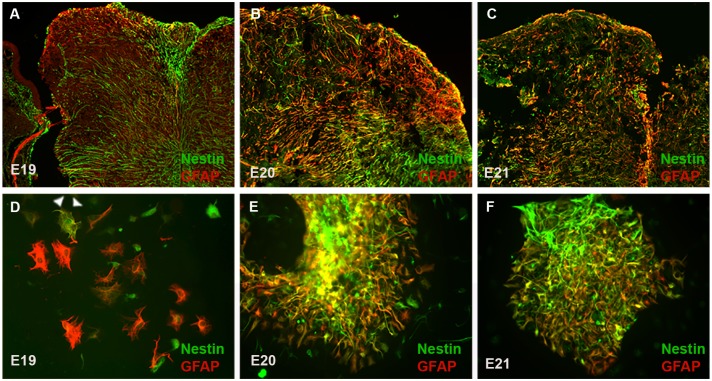
The appearance of cluster forming cells in cultures correlates with activation of astrogliosis within the spinal cord. (A, B and C) Micrographs demonstrate overlay of GFAP positive cells and nestin in sections obtained through the center of MMC lesion at E19, E20, and E21. A small population of astrocytes was identified only in the exposed dorsal area of spinal cord in MMC fetuses at E19, the intensity of GFAP staining and density of reactive astrocytes expressing nestin increased at E20, and a marked astrocytosis characterized by the presence of nestin expressing astrocytes was found throughout the exposed spinal cord tissue in MMC fetuses at E21. (Magnification 10 X). (D, E and F) Representative micrographs of MMC-AF cells 24 hour after plating demonstrate GFAP positive cells and nestin positive cells in cultures from E19, E20 and E21. (D) Double immunofluorescence staining for the expression of GFAP and Nestin shows scattered GFAP positive cells and/or nestin positive cells, which rarely co-expressed these two markers in cultures from E19. Arrowhead denotes a cell double-positive for GFAP and nestin. (E and F) Double immunofluorescence staining for the expression of GFAP and Nestin shows adherent clusters of multilayered cells containing many GFAP and nestin double positive cells in cultures from E20 and E21. (Magnification 20 X).

As described above we observed that MMC-AF cells obtained between E20 and E21 formed adherent neuroepithelial cell clusters after plating in cultures. Next, we examined whether cells from MMC-AF obtained at earlier fetal developmental stages formed similar clusters. MMC-AF cells obtained between E18 and E19 grew as a monolayer of cells and showed no cluster formation when analyzed 72 hours after plating (Fig 4A and 4B). In contrast, as described above clusters of cells were observed 24 hours after plating of MMC-AF cells from fetuses between E20 and E21 (Fig 1A–1C). The above observations show that cells forming clusters were present only in cultures from MMC-AF at the later phase of gestation. At this later stage the vulnerable exposed spinal cord of MMC fetuses is exposed to the AF and the open wound most likely allows damage and displacement of spinal tissue into the AF. Therefore, there may be a correlation between the occurrence of these cluster forming cells in AF and the astrogliosis associated with spinal cord injury. To investigate this possibility, we examined astrocytosis in the exposed spinal cord and compared it to GFAP expression in AF cell clusters obtained from MMC fetuses. Only a small population of astrocytes could be identified in dorsal area of the spinal cord in MMC fetuses at E19 (Fig 5A). The density of astrocytes expressing nestin increased at E20 when compared to E19 (Fig 5B). Furthermore, we observed a marked increase in the number of reactive astrocytes co-expressing nestin that were found throughout the exposed spinal cord tissue in MMC fetuses at E21 (Fig 5C). The abundant presence of GFAP positive astrocytes expressing nestin found within the exposed spinal cords of MMC fetuses at E20 and E21 was consistent with the occurrence of MMC-AF clusters in cultures obtained from MMC-AF between E20 and E21, but not at E19 (Fig 5). When analyzed 24–72 h after plating, double labeling for GFAP and nestin revealed only scattered GFAP positive astrocytes and/or nestin positive cells in AF cultures from E19 MMC fetuses (Fig 5D). In contrast, adherent clusters of multilayered cells containing many GFAP and nestin double positive cells were evident in MMC-AF cultures from E20 and E21 (Fig 5E and 5F). These data indicate a correlation between the presence of GFAP and nestin positive reactive astrocytes in the spinal cords of fetal rats with MMC and those present in MMC-AF clusters in cultures obtained from AF samples of MMC fetuses that appear at the later embryonic stage.

## Discussion

In this study of amniotic fluid cells from MMC-AF samples, we identified clusters of adherent cells in short-term cultures and characterized their associated phenotypic markers. These cell clusters are phenotypically reminiscent of the early neuroepithelium and they are absent in cultures from norm-AF samples. Phenotypic characterization of cells in MMC-AF clusters showed that a subset of tightly packed cells co-expressed adherens junction associated proteins ZO-1 and N-cadherin, which contribute to cell-cell adhesion characteristic of early neuroepithelial cells [36]. As previously reported, N-cadherin-based adherens junctions contribute to the strong adhesion between neuroepithelial cells during early embryonic development to maintain the structure of the neuroepithelia [40–42]. The majority of cells in MMC-AF clusters were positive for SOX-1 and Pax-6 markers of early neuroepithelial cells. Likewise, the neural stem/progenitor cell markers SOX-2 and nestin were also present in these clusters. Nestin, an intermediate filament, is transiently expressed in neuroepithelial stem/progenitor cells of the developing CNS. It is recognized as a marker for developing glial cells and neural progenitor cells and its downregulation correlates with differentiation of these cells [43]. Nestin expressing GFAP positive astrocytes were distributed throughout MMC-AF cell clusters, indicating their immature phenotype. In addition, we also found a number of mature astrocytes expressing GFAP in the absence of nestin. These findings are in line with earlier studies, which have reported the presence of GFAP positive astrocytes in a fraction of rapidly adhering cells in cultures from AF of fetuses with neural tube defects [25,26,28]. Likewise, following epigenetic selection, a sub-population of SOX-2 and nestin positive neural stem cells was isolated in culture from AF of experimental neural tube defects [29]. Our data confirm and extend on these observations, in which we show, for the first time, presence of the earliest population of neuroepithelial cells expressing ZO-1 and N-cadherin as well as SOX-1 and Pax-6 isolated from AF of MMC fetuses. We also showed that these cells exist along with SOX-2 and nestin positive cells, GFAP positive astrocytes, and doublecortin positive neurons. Thus, the data presented here combined with previous reports [29–31] shows neural cells isolated from the AF in the setting of neural tube defects contain various populations of neuroepithelial cells.

We showed that only AF samples from later gestational stages of MMC fetuses contained cells forming cell clusters phenotypically reminiscent of the early neuroepithelium. Thus, the phase of the disease was a crucial factor in the emergence of these cells into the AF. As gestation proceeds, MMC leads to injury of the openly exposed spinal cord tissue associated with significant deterioration of neurological function in MMC fetuses [15–17,44,45]. Our observation for the emergence of these clusters starting at E20 correlates with spinal cord injury in MMC fetuses and associated pathology highlighted by activation of astrogliosis at the MMC site. Astrogliosis is a common reaction in the central nervous system in response to tissue injury [46–48]. It has been shown that the expression profile of reactive astrocytes comprise early neuroepithelial markers such as nestin [46–49]. Consistent with the presence of GFAP and nestin co-expressing cells in cultures obtained from MMC-AF between E20 and E21, reactive astrocytes that also expressed nestin were found throughout the exposed spinal cord tissue in MMC fetuses at E20 and E21. Indeed, a prominent sign of astrogliosis is a marked increase in the number of GFAP positive reactive astrocytes co-expressing nestin that actively proliferate and migrate [47]. Thus, our data gives support to the hypothesis that cells in MMC-AF clusters may represent reactive astrocytes that come from the injured spinal cords directly exposed to the AF and continue to proliferate in the AF as neuroepithelial stem/progenitor cells. Mesenchymal stem cells are known to be naturally occurring in the AF during gestation [35]. It was recently shown that surgical induction of MMC in the fetal sheep model promotes stem cell phenotype and proliferation of mesenchymal stem cells isolated from the amniotic fluid [50]. Moreover, other studies reported the isolation of a significant population SOX-2 and nestin positive neural stem cells from the AF of experimental neural tube defects [29]. Although much remains to be learned, these findings suggest that AF in the MMC provides a permissive environment for proliferation of stem/progenitor cells. As mentioned above, most of neural damage observed in MMC occurs later during gestation [15–17,44,45], but, the mechanisms underlying injury to the exposed spinal cord tissue are largely unknown. Along that notion, cells growing in MMC-AF clusters may constitute a relevant and crucial *in vitro* model for studying the mechanisms of MMC development, especially the initiation and progression of spinal cord injury, in order to design novel regenerative strategies toward a better repair of MMC.

The AF provides minimally invasive access to embryonic and fetal stem cells making it an easily accessible and important source of cells for the diagnosis or therapy of developmental defects [24]. Accordingly, AF-derived cells have emerged as a potential supportive strategy for prenatal diagnosis of neural tube defects. In studies of AF cells in neural tube defects, the emphasis was on detection of neural cells from AF and more recently the impact of gestational age on relative proportions of select populations of AF cells [31]. In our studies, we showed the presence of unique cluster-forming population of neuroepithelial cells in the AF of MMC fetuses at stages pathologically associated with spinal cord injury assessed by immunohistochemical examination of astrogliosis. Thus, appearance of these cells in the AF may represent specific stage of MMC progression during fetal life and their presence may be a sensitive indicator of spinal cord damage during MMC development. Secondary *in utero* injury to the exposed spinal cord leading to astrogliosis during the later phase of gestation can disrupt normal spinal cord development that is clinically associated with deterioration of neurological function in MMC fetuses [45]. Therefore, a potential use of these cells in AF samples from MMC fetuses as a supportive diagnostic marker sensitive to the severity of spinal cord pathology may have important clinical significance.

In this study, we isolated cells from AF samples and morphologically and phenotypically characterized in short-term cultures. This method enabled us to isolate unique cluster-forming cells from the AF in the setting of MMC; however one limitation of this method is that the phenotype of isolated cells could have been affected by the exposure to culture media. Analysis of cells from fresh AF specimens using flow cytometry would overcome these limitations. Recent studies show that targeted amniotic cell profiling by flow cytometry may be a useful tool in the prenatal diagnosis and management of congenital anomalies [30]. We isolated cluster-forming cells from MMC-AF in culture and identified their associated markers. In the future, quantitative analysis of these cells from fresh AF specimens by flow cytometry should help to further elucidate the relationship between the severity of MMC and changes in the cellular content of AF at different time points during gestation.

Currently, treatment of MMC relies on surgical closure of this defect after, or in select cases, before birth [51]. Recently, a non-surgical cell-based approach as well as a scaffold-based tissue engineering approach has emerged as promising therapeutic strategies for prenatal closure of MMC lesion [23,52–57]. The idea is to provide coverage in the neonatal period for the unprotected spinal cord in order to minimize spinal cord damage at the lesion site before birth. As discussed above, the existence of neural cells in AF in the presence of MMC is most likely related to the direct contact of the vulnerable spinal cord with AF. Thus, the abundant presence of neural cells in AF at the end of pregnancy also raises the question as to whether the lack of these cells in the AF of treated fetuses can serve as a marker of therapeutic success since there are no standards to compare new treatments as they are developed.

In conclusion, we used AF as an easily accessible and important fetal environment to examine the cells associated with the development of MMC. In this study, we report that AF samples from fetal rats with retinoic acid-induced MMC yield MMC-AF clusters containing cells with a phenotype resembling neuroepithelial cells present in early stages of neural development. These cells exist in AF from fetuses with MMC, but not in the normal control fetuses. Thus, the cellular content of AF in MMC fetuses significantly differs from their normal counterparts. Even more importantly, we demonstrated that the appearance of MMC-AF clusters in cultures from the AF is related to the pathology of MMC characterized by astrogliosis in the exposed spinal cord of age matched fetuses with MMC. While the mechanisms underlying the initiation and progression of astrocytosis in the injured spinal cord of MMC fetuses remain to be determined, this work may aid in developing of methods for prenatal evaluation of MMC related spinal cord injury as well as repair of MMC lesion *in vivo* by ultimately creating therapies directed at rebuilding the fetal environment.
